# A dystrophic Duchenne mouse model for testing human antisense oligonucleotides

**DOI:** 10.1371/journal.pone.0193289

**Published:** 2018-02-21

**Authors:** Marcel Veltrop, Laura van Vliet, Margriet Hulsker, Jill Claassens, Conny Brouwers, Cor Breukel, Jos van der Kaa, Margot M. Linssen, Johan T. den Dunnen, Sjef Verbeek, Annemieke Aartsma-Rus, Maaike van Putten

**Affiliations:** 1 Department of Human Genetics, Leiden University Medical Center, Leiden, RC, the Netherlands; 2 Transgenic Facility, Leiden University Medical Center, Leiden, RC, the Netherlands; 3 Department of Clinical Genetics, Leiden University Medical Center, Leiden, RC, the Netherlands; University of Minnesota Medical Center, UNITED STATES

## Abstract

Duchenne muscular dystrophy (DMD) is a severe muscle-wasting disease generally caused by reading frame disrupting mutations in the *DMD* gene resulting in loss of functional dystrophin protein. The reading frame can be restored by antisense oligonucleotide (AON)-mediated exon skipping, allowing production of internally deleted, but partially functional dystrophin proteins as found in the less severe Becker muscular dystrophy. Due to genetic variation between species, mouse models with mutations in the murine genes are of limited use to test and further optimize human specific AONs *in vivo*. To address this we have generated the del52hDMD/*mdx* mouse. This model carries both murine and human *DMD* genes. However, mouse dystrophin expression is abolished due to a stop mutation in exon 23, while the expression of human dystrophin is abolished due to a deletion of exon 52. The del52hDMD/*mdx* model, like *mdx*, shows signs of muscle dystrophy on a histological level and phenotypically mild functional impairment. Local administration of human specific vivo morpholinos induces exon skipping and dystrophin restoration in these mice. Depending on the number of mismatches, occasional skipping of the murine *Dmd* gene, albeit at low levels, could be observed. Unlike previous models, the del52hDMD/*mdx* model enables the *in vivo* analysis of human specific AONs targeting exon 51 or exon 53 on RNA and protein level and muscle quality and function. Therefore, it will be a valuable tool for optimizing human specific AONs and genome editing approaches for DMD.

## Introduction

Duchenne muscular dystrophy (DMD) is a muscle-wasting disease with an incidence of 1:5000 new-born males [1, 2]. It is characterized by a lack of functional dystrophin resulting in continuous muscle damage, impaired regeneration and muscle tissue replacement by fibrotic and adipose tissues [3–7]. Consequently, patients progressively lose muscle function, become wheelchair dependent in early adolescence and need assisted ventilation around the age of 18. In the Western world, most patients die in their 3^rd^ or 4^th^ decade.

Research groups are exploring various therapeutic approaches to slow down disease progression. One of the approaches aiming at restoring the synthesis of the missing protein is antisense oligonucleotide (AON)-mediated exon skipping. The rationale of the exon skipping approach is based on the genetic difference between DMD and Becker muscular dystrophy (BMD) patients. In DMD patients the reading frame of dystrophin mRNA is disrupted resulting in prematurely truncated, non-functional dystrophin proteins. BMD patients have mutations in the *DMD* gene that maintain the reading frame allowing the production of internally deleted, but partially functional dystrophins leading to milder disease symptoms compared to DMD patients [8–10]. Exon skipping utilizes AONs to prevent recognition of an exon by the splicing machinery so that it is spliced out together with its flanking introns. Exons are selected for targeting in such a way that skipping them restores the reading frame, allowing the production of internally deleted but partially functional dystrophin proteins [11, 12].

Exon skipping is a mutation specific approach; different exons need to be skipped to restore the reading frame depending on the size and location of the mutation. Since most patients carry deletions and these cluster in a hotspot, skipping certain exons applies to larger groups of patients. The skipping of exon 51 would apply to the largest group (13–14%) [13, 14] and AONs targeting this exon are developed furthest. Recently, the Food and Drug Administration (FDA, USA) has granted accelerated approval to eteplirsen, an exon 51 skipping AON developed by Sarepta Therapeutics. The approval was based on minor increases in dystrophin levels in muscle biopsies from treated patients. FDA stated that as yet it has not been shown that eteplirsen also has an effect on muscle function and has requested that data to confirm this are collected by 2021 [15, 16]. Drisapersen, another exon 51 targeting AON with different chemical modifications developed by BioMarin, was deemed not ready for approval in its present form by the FDA. BioMarin withdrew their marketing application with the European Medicines Agency (EMA) and announced the discontinuation of the clinical development of not only drisapersen but also their exon 44, exon 45 and exon 53 targeting AONs [15]. Currently, in addition to the eteplirsen trials requested by FDA, trials for exon 45 and exon 53 skipping (Nippon Shinyaku and Sarepta Therapeutics) AONs are ongoing.

The most commonly used mouse model for DMD is the *mdx* mouse, which carries a spontaneous nonsense mutation in exon 23 of the *Dmd* gene [17]. Since this exon is in frame, exon 23 skipping bypasses the mutation without disrupting the reading frame. As such it can restore dystrophin production [18, 19]. While this model has been useful to show proof-of-concept of the exon skipping approach *in vivo*, it does not allow testing human specific AONs, since generally one or more mismatches occur between human and mouse target sequences. To solve this, we have developed a transgenic mouse model expressing the human *DMD* gene (B6.DBA2.129-hDMD^tg/tg^) [20]. In addition we have crossed this mouse into a mouse dystrophin negative background (B6.DBA2.129-hDMD^tg/tg^/LUMC*B10-Dmd^*mdx*^/J hDMD/*mdx*) [21] (from now on referred to as hDMD and hDMD/*mdx* mouse respectively). However, a drawback of these models is that neither has a dystrophic phenotype, due to the expression of human dystrophin [20]. Thus, while human AON-mediated exon skipping can be assessed on RNA level in these models, it is not possible to study the effects of restoration of human dystrophin protein on muscle function and quality. Obviously, a mouse model with a mutated version of the *hDMD* gene in a mouse dystrophin negative background would allow more detailed pre-clinical tests with human specific AONs. Here we present the del52hDMD/*mdx* mouse, which was generated by deleting exon 52 of the *hDMD* gene in an hDMD/*mdx* ES cell line [21] using a targeting vector and Transcription Activator-like Effector Nucleases (TALENs). Lacking both human and mouse dystrophin, these mice suffer from muscular dystrophy, as is evident from their impaired muscle function, elevated plasma creatine kinase levels, and histopathology showing centralized nuclei, fibrosis and inflammation. We also show that treatment with human specific AONs targeting exon 51 or 53 induces exon skipping and dystrophin restoration in the del52hDMD/*mdx* mouse model upon intramuscular delivery, confirming its applicability for detailed pre-clinical studies.

## Material and methods

### Mice

C57BL/6J mice were obtained from Charles River (Belgium), whereas both hDMD/*mdx* and *mdx*(BL6) mice were bred in house at the Leiden University Medical Center (LUMC) animal facility [21]. Mice were housed in IVC cages at 20.5°C with 12 hour dark-light cycles and fed regular RM3 chow (SDS, Essex, UK) *ad libitum*. All animal handling and experimentation was approved by the animal ethical committee (DEC 14180) of the LUMC. Care was taken to limit the burden and distress for the animals as much as possible.

### PCR and sequence primers

PCR and sequencing primers used are provided in S1 Table.

### Generation of the targeting construct to generate a deletion of exon 52 of the *hDMD* gene

The targeting vector (pGem-del52) was built on the backbone of the pGEM-T-easy vector (Promega Benelux, Leiden, the Netherlands). The targeting arms were generated by PCR using genomic DNA of the hDMD/*mdx* mouse ES cell line containing the complete *hDMD* gene as template [21]. Intron 51 and 52 targeting arms were 3040 and 3134 bp long respectively. The negative selection marker herpes simplex virus thymidine kinase (HSV TK) was isolated from the pKO-SelectTK plasmid whereas the positive selection marker blasticidin, flanked by LoxP sites, was isolated from the pSVBsdX1 plasmid (kind gift of A.F. Stewart, Genomics, Biotechnology Center, Technische Universität Dresden, BioInnovationsZentrum, Germany). The targeting arms and selection markers were PCR generated using the Expand Long Template PCR system of Roche (Almere, the Netherlands) (**Panel A in S1 Fig**). To facilitate cloning of the PCR isolated units, restriction enzyme digestion sites were added to the primers at the 5’end. The individual units were first cloned in the pCRII-blunt TOPO vector using the Zero Blunt® TOPO® PCR Cloning Kit (Life Technologies Ltd, Bleiswijk, the Netherlands) and Sanger sequence verified prior to cloning in the destination vector.

### Building and testing of the TALENs facilitating the targeting of the *hDMD* gene

To enhance targeting of the *hDMD* gene, TALENs were built according to the system reported by Cermak *et al*. [22] (**Panel B in S1 Fig**). TALENs were designed using the TAL Effector Nucleotide Targeter 2.0 software [23] and required plasmids obtained from Addgene (Cambridge, MA, USA). In total four sets of TALEN were constructed, two on each site of exon 52 (for sequences see **S2 Table**). Functionality of the TALENs was tested by transfection of the TALEN sets in HEK293T cells (ATCC) (**S2 Fig**). DNA of these cells was analysed by melting curve analysis (MCA) and the Surveyor Mutation Detection kit (Transgenomic, Glasgow, United Kingdom) for TALEN mediated mutations. For MCA, DNA of the TALEN and control transfected cells was subjected to PCR in presence of the DNA intercalating dye LC-Green (Bioke, Leiden, the Netherlands) and PCR products were analysed using the LightScanner (Idaho Technology, Salt Lake City, USA) to assess whether the TALENs induced mutations were present. For the Surveyor-assay DNA was subjected to PCR with the same primers as for the MCA but in absence of the LC-Green dye. PCR products were treated with nuclease according to the supplier’s manuals and resolved on a 1% agarose gel.

### Targeting of the hDMD/*mdx* ES cell line

The linearized targeting construct (25 μg) was mixed with 50 μg TALEN DNA and in a 1:2 ratio mixed with Lipofectamine®2000 (Life Technologies Ltd) according to the supplier’s manual in KO-DMEM medium without supplements. hDMD*/mdx* ES cells (4*10^7^ [21]) were harvested and suspended in the transfection mix followed by incubation for 20 min at 37°C. Twenty-four hours after plating of the transfected cells on blasticidin resistant STO feeder cells, the transfection medium was changed and selection was applied using blasticidin (Life Technologies Ltd) at a concentration of 3 μg/ml medium. Another 24 hours later, negative selection was applied by adding 1.28 μg of ganciclovir/ml (Roche, Woerden, the Netherlands). Approximately eight days after transfection, colonies were picked and individually cultured on murine embryonic fibroblasts (MEF) in 96-well plates in ES-medium. Four days later cells were split over two 96-well plates and cultured for another three days. Cells of one plate were stored in freezing medium at -80°C whereas from the counter-sample DNA was isolated to identify homologous recombination.

### Detection of homologous recombination and loss of exon 52 on DNA and RNA level in targeted ES cells

Loss of exon 52 was determined by multiplex PCR using *DMD* exon 46, exon 51 and exon 52 specific primers (**S1 Table**). The primers used to amplify exon 46 and exon 51 had been used in the past in diagnostic multiplex PCRs [24, 25].

To analyse the clones that showed no band for exon 52 in more detail, genomic DNA samples were subjected to long-range PCR (LR-PCR) using primers that hybridize to the blasticidin gene and outside the targeting arms in intron 51. PCR was performed as described for isolation of the units for the targeting vector.

Deletion of exon 52 on RNA level was determined by RT-PCR analysis of RNA isolated from ES cells cultured for a week as spin embryoid bodies (EB) in medium without LIF. These EBs contain cells of all three germ layers. Of these EBs, RNA was isolated using the NucleoSpin® RNA II kit (Bioke). RT-PCR analysis was performed as described by Spitali *et al*. [26] using previously reported primers [27]. Agarose gel purified PCR products were analysed by Sanger sequencing using the exon 50 primer also applied for nested PCR.

### Generation of the del52hDMD/*mdx* mouse strain

Recombinant ES cell clones, selected by karyotyping for the presence of 40 chromosomes in more than 80% of the cells, were injected in C57BL/6Jolahsd blastocysts, which were subsequently transplanted in pseudo-pregnant foster mice [28].

Pups were screened for inheritance of the mutated *hDMD* gene by the described multiplex PCR. Chimeric mice were then crossed with our *mdx*(BL6) mice. Pups of this mating were screened for inheritance of the mutated *hDMD* gene as well as the *mdx* mutation. Inheritance of the *mdx* mutation in exon 23 of the *Dmd* gene was determined by PCR and subsequent MCA as described above for detection of TALEN functionality. Additionally, exon 75 of the *hDMD* gene, which differs one nucleotide from the murine *Dmd* gene, was pyrosequenced. Hereto, a PCR was run 3 min at 94°C, 32 cycles of 40 sec at 94°C, 40 sec at 54°C and 1 min at 72°C followed by 5 min at 72°C and 5 min at 22°C. Streptavidin sepharose beads and buffer were added to the PCR products and mixed. Then annealing buffer and pyrosequence primers were added and incubated on a VPT Pyromark Q96 ID station. Nested-PCR analysis was performed on several pups in which inheritance of the mutated *hDMD* gene was found, to confirm the presence of the majority of the *hDMD* gene in del52hDMD/*mdx* offspring.

### Determination of functional performance and creatine kinase levels

Muscle function of the del52hDMD/*mdx* mice was compared to hDMD/*mdx* and *mdx*(BL6) mice for two successive weeks starting at an age of four weeks, where the first week served for familiarization and training of mice to the tests. Per strain, five mice of mixed genders were challenged with the forelimb grip strength test, two limb and four limb hanging tests. The tests were performed as described by Aartsma-Rus *et al*. [29], according to the standardized operating procedures from the Translational Research in Europe–Assessment and Treatment of Neuromuscular Diseases (TREAT-NMD, http://www.treat-nmd.eu/research/preclinical/dmd-sops/). Fatigability of the forelimbs upon performance of the grip strength test was determined as previously described [30].

Creatine kinase (CK) levels were determined in plasma (obtained at time of sacrifice through a cut in the tail vein) using the Reflotron CK strips and the Reflotron plus machine of Roche [31].

### Validating the dystrophic phenotype of the del52hDMD/*mdx* mice

All mice used in the functional performance assays were sacrificed by cervical dislocation and the heart and quadriceps were isolated. Dystrophin levels of both muscles were analysed by Western blot. Hereto, protein lysates were generated using the MagNa Lyser (Roche) in combination with MagNa Lyser green beads tubes in lysis buffer (containing 20% SDS in 0.1 M Tris-HCl, pH 6.8) and protein yield was determined using the Pierce BCA kit (Thermo Fisher, Etten-Leur, the Netherlands). Protein lysates from a human muscle biopsy (obtained after informed consent) was used as positive control for human dystrophin. Protein samples (25 μg total protein) were resolved on a Criterion XT 3–8% Tris-Acetate gel in XT Tricine Running buffer and run as previously described [32]. Briefly, after running, samples were blotted to Nitrocellulose paper using the Trans-blot Turbo Transfer System (Biorad). Human dystrophin protein was detected using Mandys106 antibody at a 1:125 dilution (MDA monoclonal antibody resource, Prof. Glenn Morris) while the GeneTex antibody Mandys8 (Dilution 1:2000, GeneTex, California; GTX27163) was used to detect both mouse and human dystrophin protein. As a loading control α-actinin was detected using the AB72592 antibody (dilution 1:7500) of Abcam (Cambridge, England). Protein bands were visualized using the Odyssey (Westburg, Leusden, the Netherlands) after staining with the IRDye 800CW (1:5000) labelled secondary goat-anti-mouse antibody for dystrophin and IRDye 680TL (1:10,000) labelled secondary donkey-anti-rabbit antibody for α-actinin (both from Westburg).

Tissue sections were also prepared of the heart and quadriceps and stained with human specific dystrophin primary antibodies (dilution 1:50, Mandys106, MDA monoclonal antibody resource, Prof. Glenn Morris) and laminin (dilution 1:100, ab11575, Santa Cruz) and visualized with donkey-anti-rabbit Alexa 594 (dilution 1:1000, Invitrogen), using the mouse on mouse kit (MOM, Vector Laboratories). Quadriceps sections were also stained for α-sarcoglycan (dilution 1:50, NCL-α-sarco, Leica) and β-dystroglycan (dilution 1:50, sc-28535, Santa Cruz) using the same protocol. Additionally, a Haematoxylin and Eosin staining was performed as previously described [31]. The percentage of centralized nuclei in the quadriceps was manually counted for four mice per strain (2 males, 2 females) on five pictures randomly taken at a 10x magnification. The total number of myofibers counted varied between 1452 and 2833. We assessed the number of revertant fibers on an entire muscle cross-section for three mice per strain by manual counting.

### Treatment of the del52hDMD/*mdx* model with human specific exon 51 and 53 AONs

Four months old female del52hDMD/*mdx*#35 mice (n = 2) were anaesthetized with 2% isoflurane and treated intramuscularly in both gastrocnemius and triceps muscles with human specific Vivo-Morpholinos (ViM) targeting either exon 51 (5’-TCAAGGAAGATGGCATTTCTAGTTT-3’) or 53 (5’-TTGCCTCCGGTTCTGAAGGTGTTCT-3’) (40 μg in 50 μl saline) on two consecutive days. The ViMs targeted exonic regions, i.e. nucleotides 63–87 of exon 51 and nucleotides 35–59 of exon 53. The exon 51 and 53 ViMs had two and four mismatches, respectively, with the mouse sequence. Four weeks after the final injection, mice were sacrificed by cervical dislocation and muscles were snap frozen in liquid nitrogen cooled isopentane and stored at -80°C. Muscles were cut with a cryostat and sections were collected in two MagNA Lyser tubes containing zirconium beads (1.4 mm; OPS Diagnostics) such that both tubes contained tissues of all parts of the muscle.

RNA was isolated in TriSure reagent (Roche) and 400 ng was used for RT-PCR in which RNA was incubated for 30 min at 55°C with specific primers targeting exon 57 (**S1 Table**). Human specific exon 51 and 53 skipping was assessed by nested PCR analyses on RNA (20 cycles of 94°C for 40s, 60°C for 40s and 72°C for 80s) using primers specific for exon 47 and 54. 1.5 μl PCR product was used in a nested PCR (32 cycles of 94°C for 40s, 60°C for 40s and 72°C for 60s) using primers targeting exon 48 and 54. PCR products were visualized on a 1% agarose gel. Purification of PCR product and Sanger sequencing were performed as described above, using primers targeting exon 51 and 53. A murine specific nested PCR was performed on cDNA synthesized with murine specific exon 56 primers. We utilized murine specific primers targeting exon 49 and 54 for the first PCR, and exon 50 and 54 for the second PCR. The same PCR protocol was used as for the human specific nested-PCR. All PCR products were Sanger sequenced using primers targeting exon 50 and 54. Dystrophin expression was assessed by Western blot analyses as described above with some adjustments. Total protein loaded was 30 μg and membranes were incubated with Mandys106 antibody at a 1:50 dilution (MDA monoclonal antibody resource, Prof. Glenn Morris) to target human specific dystrophin protein. As a loading control α-actinin was detected using the AB72592 antibody (dilution 1:1000) of Abcam.

### Statistics

Statistical analyses were performed with GraphPad Prism version 6.02. Central nucleation, functional and CK data were analysed with a one-way ANOVA, followed by a Bonferroni post-hoc test in case of significance. For all tests a *P*<0.05 was considered significant. Data are displayed as mean ± SEM.

## Results

### Inefficient targeting of hDMD/*mdx* ES cells without TALENs

We aimed to introduce a deletion of exon 52 in the *hDMD* gene, since this would result in an animal model to test and optimize AONs targeting either exon 51 or exon 53 *in vivo*. We first attempted to generate the model using homologous recombination with a targeting vector (**Panel A in S1 Fig**). The *hDMD* gene in the hDMD/*mdx* ES cells was targeted by electroporation of the linearized targeting vector into the cells. In two independent experiments approximately 1000 colonies were picked and the 500 surviving colonies were analysed, but none were properly targeted. Reasoning that too much blasticidin might skew towards clones with multiple, random integration sites, we performed another targeting experiment with less blasticidin (3 μg/ml vs 6 μg/ml). In this experiment 500 colonies were picked but again none were properly targeted.

### TALEN facilitated targeting of hDMD/*mdx* ES cells

**Fig 1:**
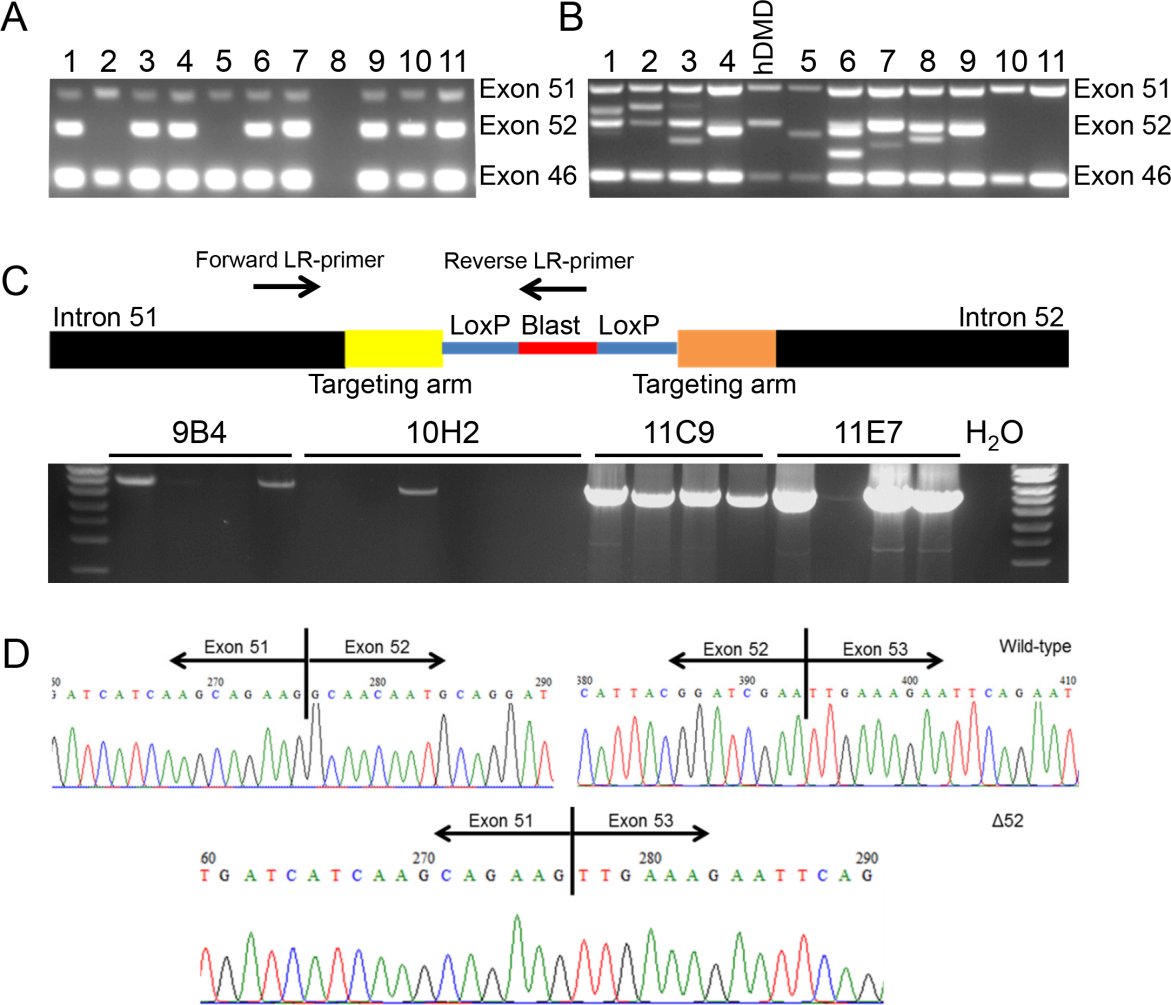
PCR analysis of targeted clones and confirmation of deletion exon 52 on RNA level. Single ES clones were cultured in 96-well plates and DNA was isolated and used as template in a multiplex PCR. Here the exons 46, 51 and 52 of the *hDMD* gene were analysed where exon 46 and 51 are positive controls and exon 52 the target to be deleted. **A**) An example is shown where candidate samples 2 and 5 are of interest because they are negative for exon 52 but positive for the control exons. **B**) For a large number of clones additional fragments were found for exon 52, suggesting non-homologues end joining (NHEJ) of TALEN induced double stranded breaks **C**) Representative image of LR-PCR performed on DNA of sub-clones of four exon 52 negative clones (9B4, 10H2, 11C9 and 11E7). LR-PCR was performed with primers targeting intron 51 (outside the targeting arm) and blasticidin (only present after homologous recombination), to rule out loss of PCR primer recognition sites by NHEJ and to confirm true targeting. **D**) RT-PCR was performed for RNA isolated from embryoid bodies of selected clones. The different fragments were isolated, purified and Sanger sequence analysed. In the wild type situation exon 52 was present, whereas in the properly targeted clones exon 52 was not present. This confirmed the exon 52 deletion on RNA level.

TALENs are able to facilitate homologous recombination by inducing double stranded breaks in the DNA at specific locations [22, 33]. Therefore we designed and built two TALENs sets with a recognition sequences close to the intron 51-exon 52 and exon 52-intron 52 boundary to facilitate the targeting of exon 52 (**Panel B in S1 Fig**). Their functionality was tested by transfecting them into HEK293T cells. Three days after transfection, DNA of the cells was isolated and subjected to MCA and Surveyor assay (**S2 Fig**), which confirmed that all four TALEN sets were functional. Since TALEN set pTAL52KL facilitated easy analysis using melting curve analysis, this set was chosen for the actual targeting experiment. We transfected hDMD/*mdx* ES cells with the linearized targeting vector together with the TALEN DNA using Lipofectamine. After targeting, 1008 colonies were picked from which 32 clones (3.2%) were negative for exon 52 (**Fig 1A**, example of multiplex PCR), while, 39 clones (3.9%) showed a PCR fragment of different length (**Fig 1B**). These clones were likely the result of non-homologous enjoining (NHEJ) of TALENs induced double stranded breaks. To rule out that the exon 52 deleted clones were in fact clones where the primer annealing site was lost due to NHEJ rather than homologous recombination, samples were further subjected to LR-PCR with one primer annealing to the blasticidin selection marker gene and one annealing to a sequence outside the targeting arm. This resulted in a PCR product of the expected length in nine clones, confirming that homologous recombination had taken place (**Fig 1C**).

In addition, we examined whether this indeed resulted in mRNA lacking exon 52. Nested PCR of the recombinant ES cell clones showed shorter fragments for the target clones. Sanger sequencing confirmed the deletion of exon 52 (**Fig 1D**). Subsequently, four sub-clones were tested for their karyotype out of which three clones (9B4, 10H2 and 11E7) met the criteria outlined in the Methods. These clones were expanded and used for the generation of del52hDMD/*mdx* mice.

### Generation of the del52hDMD/*mdx* mouse

**Fig 2:**
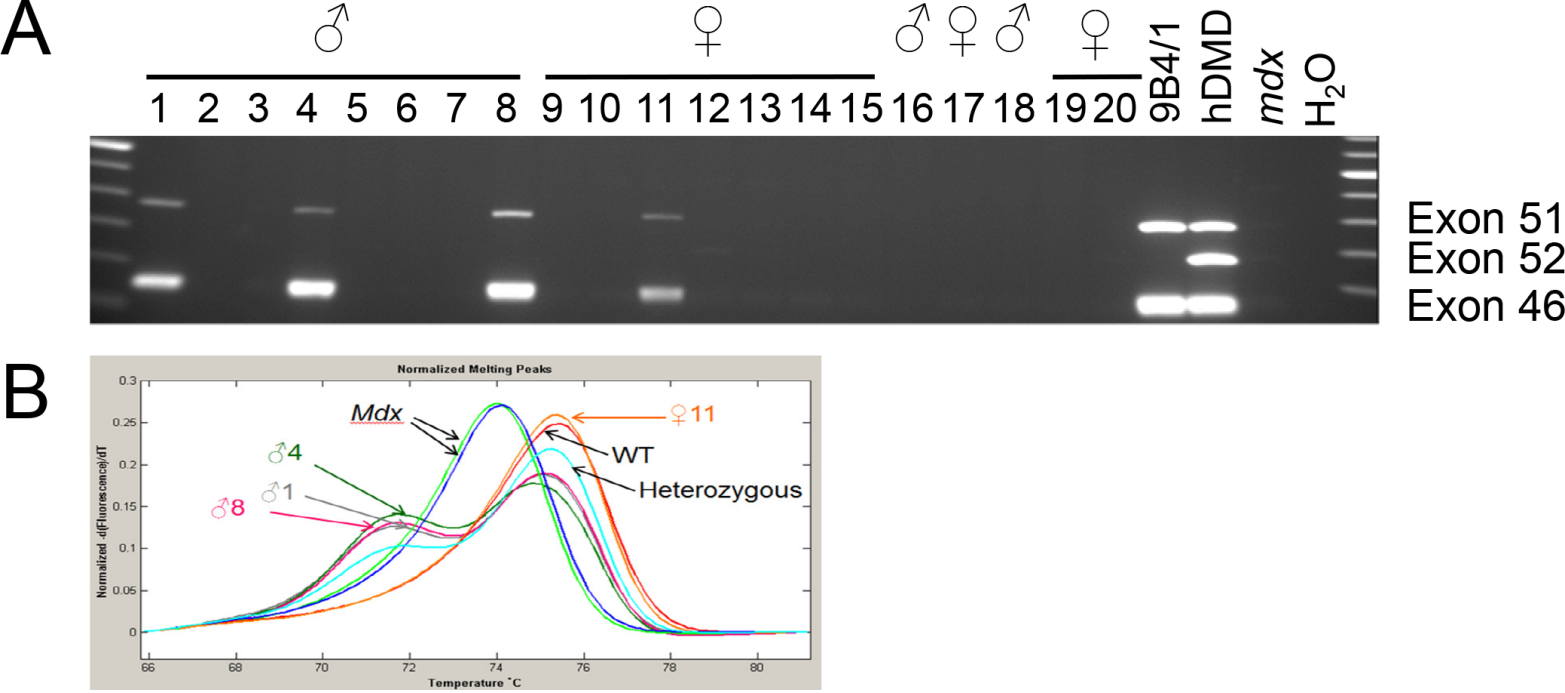
Confirmational analysis of offspring from blastocyst transplanted mice. Pups were analysed for chimerism by multiplex PCR and MCA. **A**) Four of the pups derived after transplantation of blastocysts injected with ES cells of clone 9B4 showed presence of the exon 52 deleted *hDMD* gene (lines 1, 4, 8 and 11). **B**) Melting curve analysis revealed that all male pups were also chimeric for the *mdx* point mutation.

Transplantation of the injected blastocysts resulted in 5 positive pups (4 males, 1 female out of 36 born). Mice were tested positive for exon 46 and 51 of the *hDMD* gene while lacking exon 52 (**Fig 2A, S2 Table**). Moreover, all male pups were chimeric for the *mdx* point mutation, while the female did not carry this mutation (**Fig 2B**). We confirmed expression of the complete *DMD* gene using primer pairs to generate various overlapping PCR fragments (**S3 Fig**).

Chimeric mice were mated with *mdx*(BL6) mice to generate F1 mice with the del52*hDMD* gene. All pups were screened for inheritance of the del52*hDMD* gene and three pups were found to carry this gene indicating germline transmission of the transgene. The two deletion strains were termed del52hDMD*/mdx*#35 (derived from ES cell clone 9B4) and del52hDMD*/mdx*#37 (derived from ES cell clone 11E7). The F1 mice positive for the del52*hDMD* gene were mated with *mdx*(BL6) females generating F2 mice that subsequently were inter-crossed to obtain mice being homozygous for the del52*hDMD* gene and the *mdx* point mutation.

### Characterization of dystrophin expression and pathology in the del52hDMD/*mdx* models

**Fig 3:**
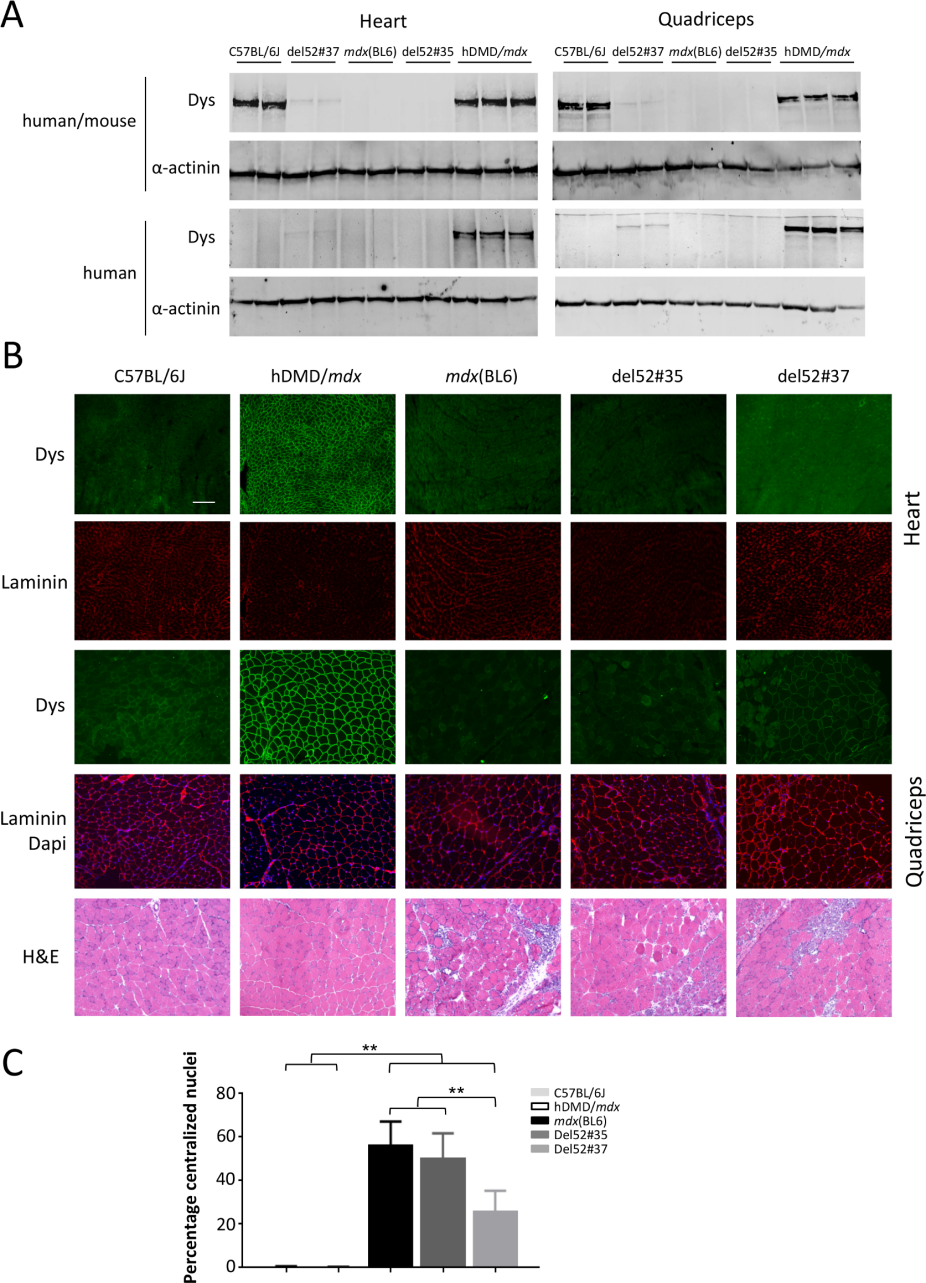
Dystrophin expression pattern and morphological examination of del52hDMD/*mdx* mouse lines. **A**) Western blot analyses of heart and quadriceps, incubated with either GTX (human and mouse specific) or Mandys106 (human specific). Wild type expression levels of human dystrophin were observed in hDMD/*mdx* mice. Notably, del52hDMD/*mdx*#37 mice expressed traces of human dystrophin, in both cardiac and skeletal muscle, while this was not observed in del52hDMD/*mdx*#35 and *mdx*(BL6) mice. **B**) Sections of the heart and quadriceps stained with human specific dystrophin antibodies. Expression of human dystrophin is at wild type level in hDMD/*mdx* mice as anticipated. Both C57BL/6J, *mdx*(BL6) and del52hDMD/*mdx*#35 mice did not express human dystrophin. Interestingly, in most fibers of del52hDMD/*mdx*#37 mice, human dystrophin was expressed at low levels. Haematoxylin and eosin staining revealed signs of degeneration and regeneration in the quadriceps of both del52hDMD/*mdx* strains, as evident by variation in fiber size, centralized nuclei and patches of fibrosis and inflammation. Overall pathology appeared to be slightly less extensive in del52hDMD/*mdx*#37 mice compared to *mdx*(BL6) and del52hDMD/*mdx*#35 mice. **C**) Almost no centralized nuclei were found in wild type mice, while half of the myofibers in *mdx*(BL6) and del52hDMD/*mdx*#35 mice had centrally located nuclei. The percentage in del52hDMD/*mdx*#37 mice was with 26% significantly lower. Data were based on manual counts of 5 randomly taken pictures of 2 males and 2 females per genotype. Asterisks indicate *P*<0.01.

Mice (n = 5 per group) were sacrificed at the age of six weeks and heart and quadriceps muscles were isolated and snap-frozen in isopentane cooled in liquid nitrogen. Dystrophin protein expression was assessed by Western blot analyses (**Fig 3A**) and immunofluorescence (**Fig 3B**). For del52hDMD*/mdx*#35 mice, no dystrophin was detected in both muscles on the Western blot, while a trace amount of dystrophin of human origin was present in del52hDMD*/mdx*#37 samples. Notably, del52hDMD/*mdx*#37 mice indeed expressed dystrophin in very low amounts in all their myofibers, while del52hDMD/*mdx*#35 mice did not. Occasional revertant fibers were observed in the quadriceps of mice from the three dystrophic strains (**Panel A in S4 Fig**). Expression levels of α-sarcoglycan and β-dystroglycan were decreased in the dystrophic strains (**Panel B in S4 Fig**). To characterize overall pathology, cross sections of the quadriceps were stained with haematoxylin and eosin. Muscle pathology consisted of cycles of degeneration and regeneration, as evidenced by the high number of small, centrally nucleated fibers, fibrotic lesions and inflammatory cells. The percentage of centralized nuclei in *mdx*(BL6) and del52hDMD/*mdx*#35 mice varied between 56 and 50% respectively (**Fig 3C**). The percentage of central nucleation in the del52hDMD/*mdx*#37 mice was significantly lower (26%), most likely due to the expression of low dystrophin levels.

### Functional performance of the del52hDMD/*mdx* mice

Four week old hDMD/*mdx*, *mdx*(BL6), del52hDMD*/mdx*#35 and del52hDMD*/mdx*#37 mice were functionally tested for two consecutive weeks, where the first week served to familiarize and train the animals. Groups contained five mice of mixed genders. The weight of the mice at start of the experiment was approximately 15 grams independent of the strain. After two weeks of functional testing all mice, regardless of the strain, had gained on average 4 grams. However, as anticipated in all groups and at any time, male mice were heavier than female mice.

**Fig 4:**
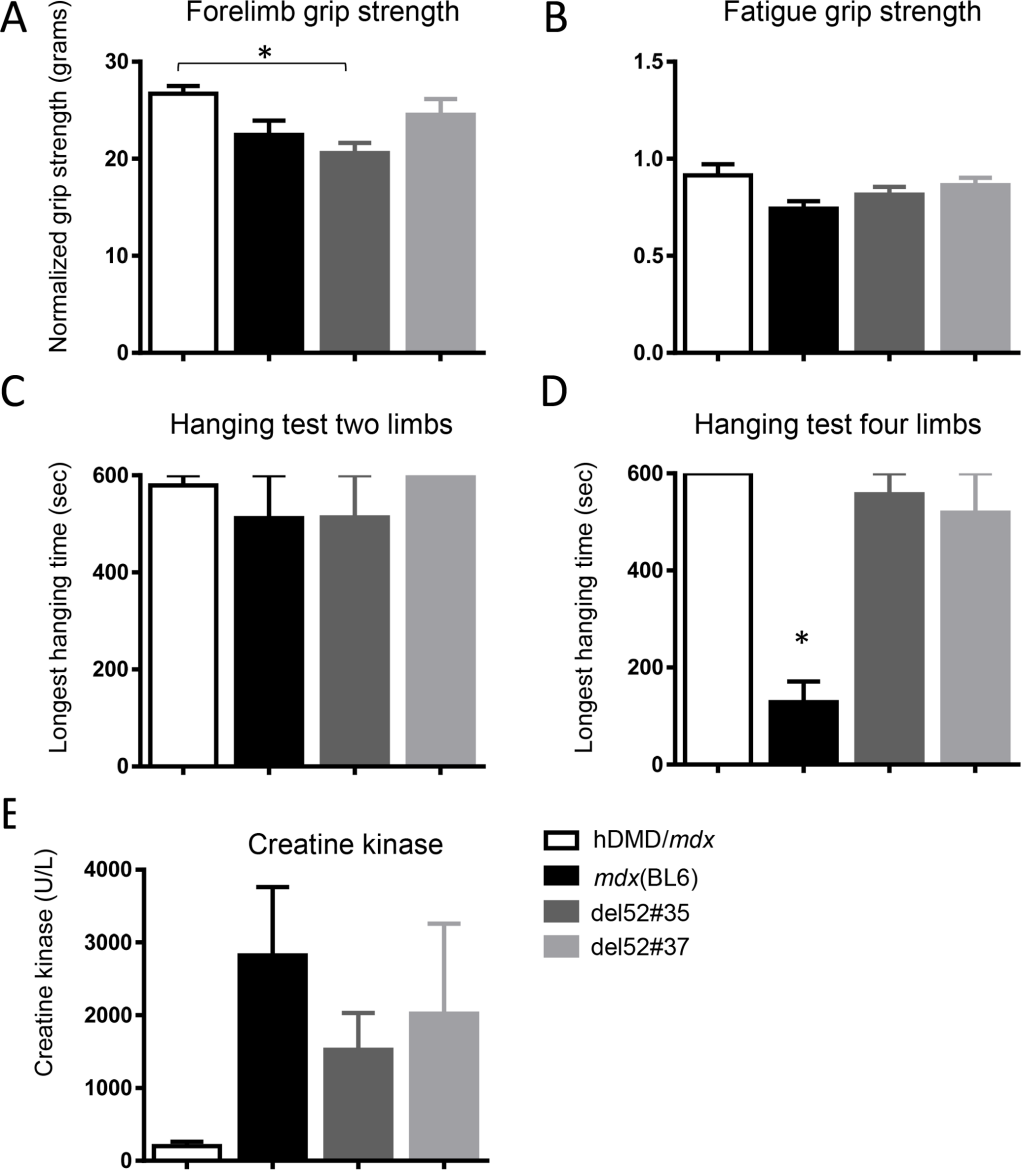
Functional performance is impaired in del52hDMD/*mdx* mice. **A**) Forelimb grip strength was impaired in *mdx*(BL6) and del52hDMD*/mdx* mice. **B**) hDMD/*mdx* mice were resistant against fatigue, while muscles of *mdx*(BL6) and del52hDMD*/mdx* mice were fatigued at the end of the grip strength protocol. Performance in hanging tests starting with two **C**) and four limbs **D**) was impaired in *mdx*(BL6) and del52hDMD*/mdx* mice. Overall, del52hDMD*/mdx*#37 mice outperformed *mdx*(BL6) and del52hDMD*/mdx*#35 mice. **E**) Creatine kinase levels were elevated in both del52hDMD*/mdx* and *mdx*(BL6) strains compared to hDMD/*mdx* mice. Asterisk indicates *P*<0.05, data are represented as the mean ± SEM. hDMD/*mdx*, *mdx*(BL6) and del52hDMD*/mdx*#35 groups consisted of n = 3 males and n = 2 females (one male *mdx*(BL6) mouse died), while the del52hDMD*/mdx*#37 group consisted of n = 2 males and n = 3 females.

The functional performance of the mice was determined with the forelimb grip strength test, and two and four limb hanging tests. For dystrophin negative *mdx*(BL6) and del52hDMD*/mdx*#35 mice, grip strength was impaired compared to hDMD/*mdx* mice, while del52hDMD*/mdx*#37 mice performed slightly better (**Fig 4A**). Both deletion 52 strains were more resistant to fatigue than *mdx*(BL6) mice but not to the same extent as wild type mice (**Fig 4B**). In the two and four limb hanging test, hDMD/*mdx* mice consistently reached the maximum allowed hanging time of 600 sec. In the two limb hanging test, *mdx*(BL6) and del52hDMD*/mdx*#35 mouse performance was worse than that of hDMD/*mdx* mice, but comparable to each other (**Fig 4C**). Slightly better hanging times were obtained by del52hDMD*/mdx*#37 mice, although none of these differences was significant. Performance of *mdx*(BL6) in the four limb hanging test was significantly impaired. Although both del52hDMD/*mdx* strains hung for a shorter duration, this did not reach significance. Overall, female mice outperformed males in the hanging tests, regardless of the strain. It is possible that the overall better performance of the del52hDMD*/mdx*#37 mice results from the expression of low dystrophin levels observed in these mice, or from a slightly higher number of females used in this group. Before sacrifice, blood was collected to measure plasma CK levels as a marker for muscle integrity. While CK levels were low (<500U/L) for the hDMD/*mdx* control mice, CK levels of *mdx*(BL6) mice were 14 times higher (**Fig 4E**). Also mice of the two del52hDMD*/mdx* strains had elevated CK levels being approximately 10 times higher compared to hDMD/*mdx* mice.

### Local treatment of AONs targeting exon 51 and 53 results in exon skipping and dystrophin synthesis

Two del52hDMD*/mdx*#35 mice were intramuscularly treated in the gastrocnemius and triceps with human specific Vivo morpholinos (ViMs) targeting either exon 51 or 53 on two consecutive days and exon skipping and dystrophin expression were assessed four weeks after the last injection.

**Fig 5:**
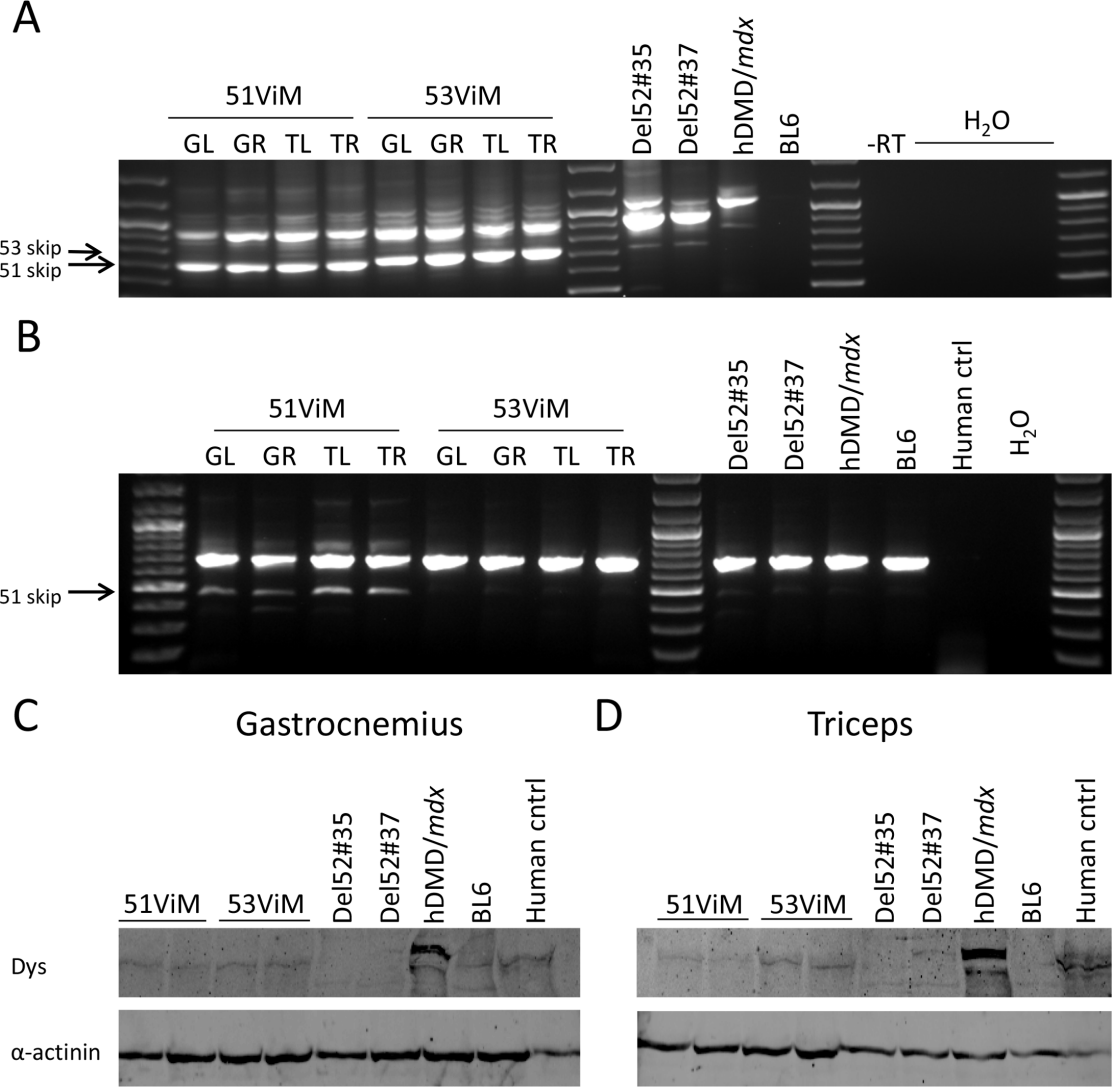
ViM treatment results in exon skipping and subsequent dystrophin restoration. **A**) Nested PCR revealing exon skipping upon local 51ViM or 53ViM treatment in right and left gastrocnemius and triceps muscle (respectively GR, GL and TR, TL) of two del52hDMD*/mdx*#35 mice. We confirmed by Sanger sequencing that the upper band in the untreated del52hDMD*/mdx* samples involves a cryptic splicing event that is occasionally observed in untreated mice of this strain. It contains exon 51, part of intron 51, the last 101 nucleotides of exon 52, exon 53 and multiple stop codons. The arrows indicate the expected heights of fragments lacking exon 51 (700 bp) or exon 53 (721 bp). **B**) Murine specific nested PCR confirmed that the exon 51 ViM induced low levels of mouse exon 51 skipping. The exon 53 ViM only resulted in exon 53 skipping in the human transcript as no skipping band was seen in the PCR performed with mouse-specific primers (expected size 483 bp). Sanger sequence confirmed that the smaller fragment obtained in the ViM exon 51 treated muscles contained the boundary of exon 50–52. Human ctrl; healthy human control sample. The arrow indicates the expected height of fragments lacking exon 51 (463 bp). **C-D**) Exon skipping resulted in the restored dystrophin expression in gastrocnemius (C) and triceps (D) of 51ViM and 53ViM treated mice.

Local treatment with exon 51 or 53 specific AONs in del52hDMD*/mdx*#35 mice resulted in pronounced exon skipping in both gastrocnemius and triceps muscles (**Fig 5A**). We also performed a mouse specific PCR to study potential exon skipping in murine dystrophin transcripts. While the ViMs targeting exon 53 did not induce skipping of murine exon 53, the exon 51 specific ViMs did also induce mouse exon 51 skipping albeit at much lower levels than observed in the human transcript (**Fig 5B**). Reading frame restoration resulted in synthesis of human specific dystrophin protein as shown in **Fig 5C and 5D**.

## Discussion

The *mdx* mouse model has been extensively used for pre-clinical evaluation of AONs. However, this model has a mutation that lies upstream of the human deletion hotspot (exon 45–55) for DMD and BMD patients. The *mdx*52 model has a deletion of exon 52 in the mouse *Dmd* gene [34] and has been extensively used to test mouse specific AONs [35–37]. Although it resembles the human situation better than the *mdx* model it cannot be used to test human-specific AON sequences. To allow direct testing of human specific AONs we have constructed the del52hDMD*/mdx* mouse, which carries a deletion of exon 52 in the human *DMD* gene while lacking a functional murine *Dmd* gene. Since AONs are sequence-specific, the del52hDMD*/mdx* mouse has the advantage over the *mdx*52 mouse, in that human-specific AONs can now for the first time be pre-clinically tested in a humanized, dystrophic mouse model. We here show in a small proof-of-concept study that treating del52hDMD*/mdx#*35 mice with human specific AONs indeed restored the disrupted open reading-frame, thereby allowing synthesis of truncated dystrophin protein. Our exon 51 specific ViM also induced mouse exon 51 skipping despite the two mismatches. Skipping levels of murine origin were however lower than of human origin. This is in line with a previous study in which hDMD mice were treated with either phosphorodiamidate morpholino oligomer (PMO) or 2′-O-methyl phosphorothioate (2OMePS) AONs with the identical AON sequence of the ViM used here [38]. Heemskerk *et al*. observed that 2OMePS AONs did not induce skipping of the murine exon 51, while PMOs did. The exon 53 ViM, which had four mismatches distributed over the AON sequence, did not induce skipping of the murine exon 53. These findings indicate that the AON chemistry and number of mismatches can influence aspecific targeting and highlight the importance of testing skipping of the murine *Dmd* gene when using the del52hDMD/*mdx* mouse. Notably, skipping exons 51 or 53 of the murine *Dmd* will not restore mouse dystrophin expression, but will lower the amount of AONs available for targeting of the human *DMD* gene. More in-depth longitudinal studies including additional analyses, such as electrophysiological tests, should be performed to draw conclusions on compound efficacy and safety.

Clinical trials investigating AONs targeting exon 51 and 53 are ongoing. Nevertheless, our del52hDMD/*mdx* mouse has added value as it allows answering questions that arise from these clinical studies but cannot be easily answered in human trials (e.g. detailed study of AON uptake and pharmacokinetics and pharmacodynamics in all skeletal muscles). Furthermore, it is clear there is still room for improvement with regards to AON efficiency and this model enables pre-clinical testing of new AON chemistries but also genome editing approaches on mRNA, protein and functional levels. This model also offers the unique opportunity to test multiple exon skipping approaches such as exon 45–55 skipping. Feasibility for this approach has already been tested in the *mdx*52 model [36, 37], however the del52hDMD/*mdx* model allows multiple exon skipping with human specific sequences.

It was rather challenging to generate the exon 52 deletion in the hDMD/*mdx* ES cell line. This was surprising because the *DMD* gene is frequently mutated in human germline (one in three DMD cases carries a *de novo* mutation). Using TALENs we were able to generate the intended mutation, in line with previous reports of TALEN facilitated homologous recombination [39]. Deletion of exon 52 caused dystrophinopathy in del52hDMD/*mdx* mice leading to characteristic, *mdx* like, histopathological features (i.e. fibrosis, inflammation, degeneration and regeneration) and impaired muscle function. Further studies are needed to assess skeletal muscle and heart pathology and function in more detail and over a longer time course.

Notably, del52hDMD/*mdx*#37 mice were less severely affected than del52hDMD/*mdx*#35 mice, possibly due to the trace dystrophin expression observed by Western blot and immunofluorescence in both heart and quadriceps of del52hDMD/*mdx*#37 but not of del52hDMD/*mdx*#35 mice. This is in line with our previous observations in *mdx-Xist*^*Δ*hs^ and *mdx-utrn*^*-/—*^*Xist*^*Δ*hs^ mice where a few dystrophin positive fibers (resulting from skewed X-inactivation) also led to less severe muscle pathology and function [40, 41]. The trace levels of dystrophin in the del52hDMD/*mdx*#37 line make these mice less applicable for genetic therapy studies. However, the model offers interesting possibilities for following the effect of low dystrophin levels.

Since AON-mediated exon skipping is a mutation specific approach, multiple AONs targeting different exons are needed. To allow testing and optimization of other human AONs, additional hDMD deletion models are required. The CRISPR/Cas9 site-specific genome editing system [42] could be instrumental in generating these. In fact, we managed to obtain an hDMD/*mdx* ES line with a deletion of exon 45 using this technology, suggesting that CRISPR/Cas9 is an easy and straightforward way to generate additional animal models. This is underlined by a recent publication from Young *et al*. who generated an exon 45 deletion in the hDMD/D2-*mdx* model through CRISPR/Cas9 injection into hDMD zygotes [43]. Further functional analysis of this model is pending, but as pointed out by the authors, once validated, this model would allow assessment of exon 44 and exon 46 skipping AONs.

In conclusion, the *DMD* gene present in the hDMD/*mdx* ES cells can be modified using state of the art gene editing systems such as TALENs and CRISPR/Cas9. Using such a system, we now have generated the del52hDMD/*mdx* mouse. This is the first pre-clinical animal model that allows testing of human AONs that mediate skipping of either exon 51 or 53, both resulting in an in-frame mRNA. Their efficacy cannot only be analysed on mRNA and protein level but also on the level of muscle integrity and function.

## Supporting information

S1 ARRIVE Checklist This file contains ARRIVE (animal research: Reporting of *in vivo* experiments) guidelines checklist that is required for reporting of research using animals. (DOCX)

S1 Table Primers used for PCR reactions. Primers for generating DNA used for cloning were constructed with an additional restriction side as indicated. ^a^ marked primers were used in melting curve analysis. Those primers marked with ^b^ were used in multiplex PCR reactions (product size of exon 46, 51 and 52 were respectively 148 bp, 388 bp and 273 bp). The primer set marked by ^C^ was developed by Beggs [25] and the ^d^ marked set by Chamberlain [24].
(DOCX)

S2 Table Sequences of TALENs sets and number of chimeric pups that arose from blastocyst injections. (DOCX)

S1 Fig Schematic representation of the targeting vector and TALENs facilitated targeting. **A**) Schematic representation of the targeting vector used to delete exon 52 of the *hDMD* gene. HSV-TK is the negative selection marker, which is placed outside the targeting arm area. I51 and I52 are the targeting arms consisting of autologous *hDMD* DNA sequence of the intron 51 and 52 obtained by LR-PCR. In between the targeting arms the gene of the positive selection marker blasticidin (blast) is placed, which is flanked by LoxP sites. **B**) Schematic representation of TALENs facilitated targeting of the *hDMD* gene. The recognition site of the TALEN set pTal52K/L is at the intron 51 exon 52 boundary and is close to the intron 51 targeting arm. The TALENs induce a double strand break and upon homologous recombination with the targeting vector exon 52 is replaced by the blasticidin gene (NB blasticidin expression is in the opposite direction as dystrophin).
(PDF)

S2 Fig Determining functionality of the TALENs. Functionality of the TALENs was determined by analysing DNA of HEK293T cells transfected with the TALENs for TALEN mediated double strand breaks followed by NHEJ. The target area of the TALENs was PCR amplified using melting curve analysis (MCA) **A**) or **B**) surveyor assay. The PCR product was analysed for mutations in the sequence, and compared to PCR product utilizing wild type DNA as PCR-template. Data revealed that pTAL-52 GH and KL were most functional with pTAL52KL having the highest functionality (based on MCA). Therefore, the latter set was chosen to facilitate the targeting.
(PDF)

S3 Fig Nested-PCR confirming the presence of the majority of the *hDMD* gene. The nested-PCR products have the following sizes: ex 11–15; 633 bp, ex 19–25; 1016 bp, ex 27–30; 463 bp, ex 31–35; 678 bp, ex 43–48; 805 bp, ex 48–53; 735 bp (without exon 52; 617 bp); ex 58–62; 621 bp; ex 62–68; 744 bp.
(PDF)

S4 Fig Expression of several dystrophin-glycoprotein complex proteins. **A**) The number of revertant fibers, counted on the whole cross-sectional area of the quadriceps muscle, was similar between the three dystrophic mouse strains. **B**) The quadriceps of all strains stained for dystrophin, α-sarcoglycan and β-dystroglycan. Scale bar is 100 μm.
(PDF)
