# Supplementary material for: A dystrophic Duchenne mouse model for testing human antisense oligonucleotides
Source: PLoS One. 2018 Feb 21;13(2):e0193289. doi: 10.1371/journal.pone.0193289 (PMC5821388; doi:10.1371/journal.pone.0193289)

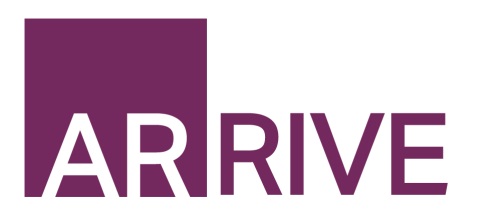


The ARRIVE Guidelines Checklist

Animal Research: Reporting In Vivo Experiments

Marcel Veltrop^1^, Laura van Vliet^1^, Margriet Hulsker^1^, Jill Claassens^2^, Conny Brouwers^2^, Cor Breukel^2^, Jos van der Kaa^2^, Margot M. Linssen^2^, Johan T. Den Dunnen^1,3^, Sjef Verbeek^1,2^, Annemieke Aartsma-Rus^1,^* and Maaike van Putten^1^

^1^Department of Human Genetics, Leiden University Medical Center, Leiden, 2300 RC, The Netherlands.

^2^Transgenic Facility, Leiden University Medical Center, Leiden, 2300 RC, The Netherlands.

^3^Department of Clinical Genetics, Leiden University Medical Center, Leiden, 2300 RC, The Netherlands.

|  | | ITEM | RECOMMENDATION | Section/ Paragraph |
| --- | --- | --- | --- | --- |
| 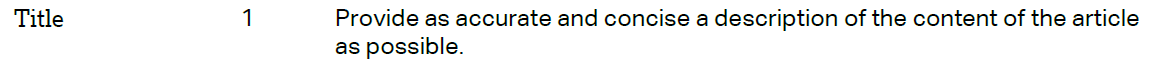 | | | Title |  |
| 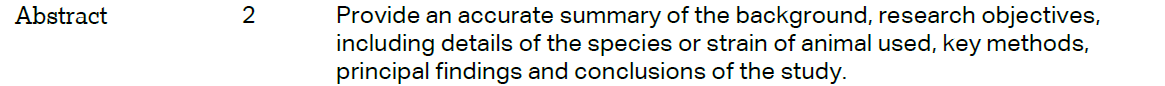 | | | Abstract |  |
| INTRODUCTION | | |  |  |
| 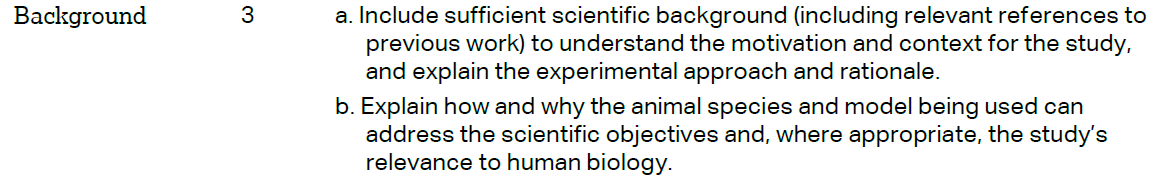 | | | Paragraphs 1-2  Paragraphs  2-3 |  |
| 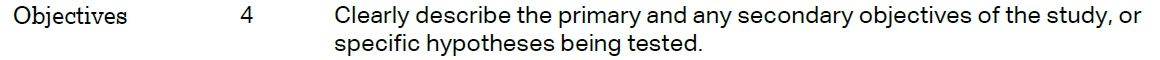 | | | Paragraph 3 |  |
| METHODS | | |  |  |
| 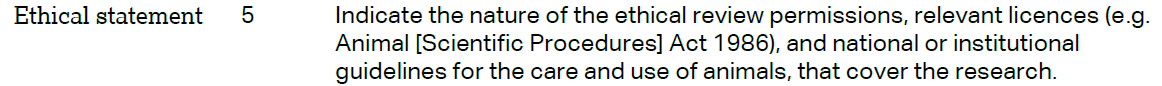 | | | Section 1 |  |
| 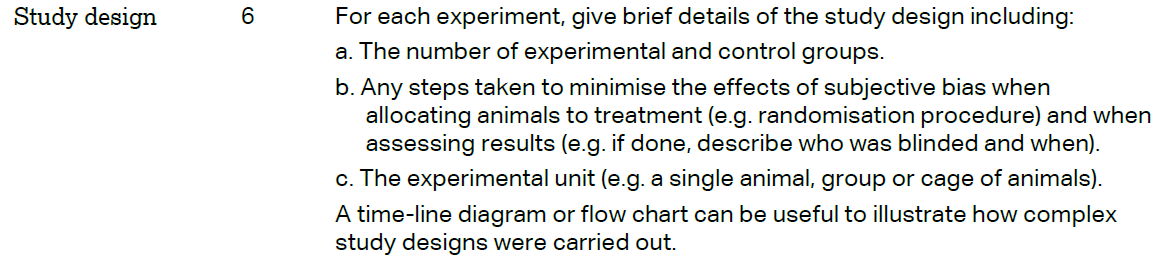 | | | Section 8, 10  Sections  1-2  Section 1 |  |
| 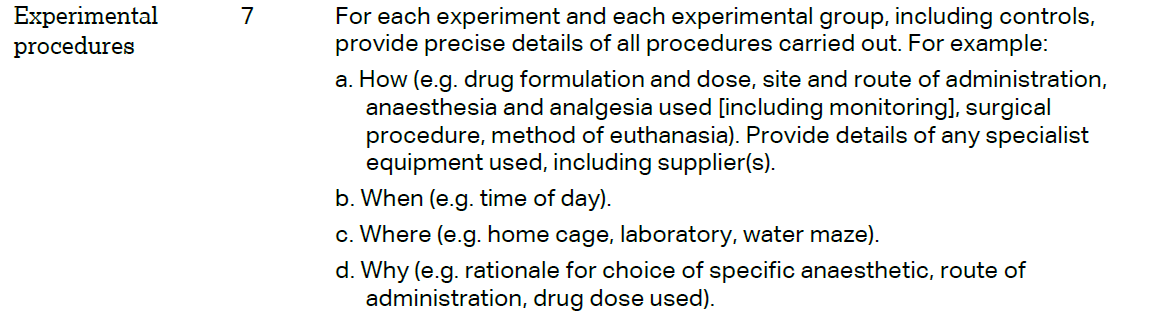 | | | Sections  1, 8-10 |  |
| 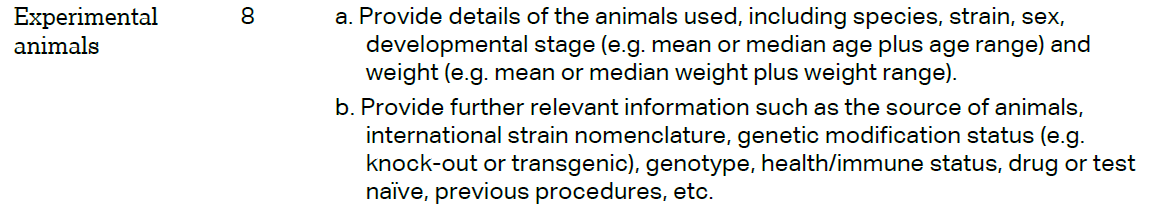 | | | Sections  1, 9-10  Section 1 |  |

The ARRIVE guidelines. Originally published in *PLoS Biology*, June 2010^1^

| 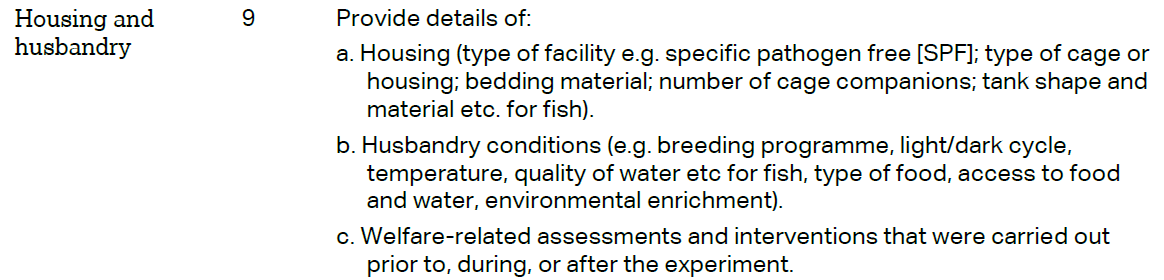 | Section 1  Section 1  Section 1 | |
| --- | --- | --- |
| 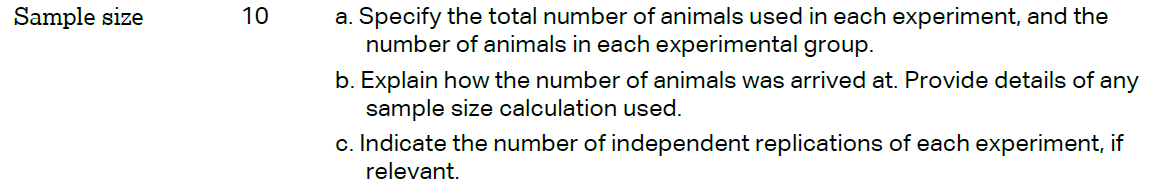 | Section 1, 9-10 | |
| 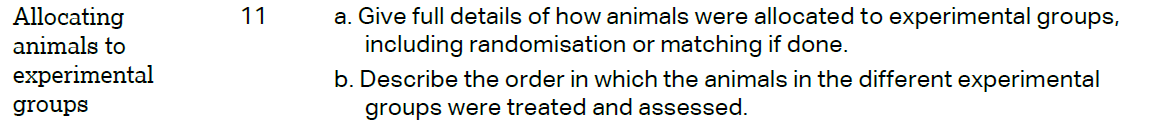 | Section 1, 9-10 | |
| 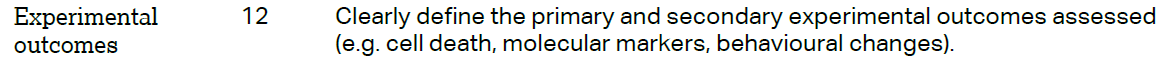 | Section 9-10 | |
| 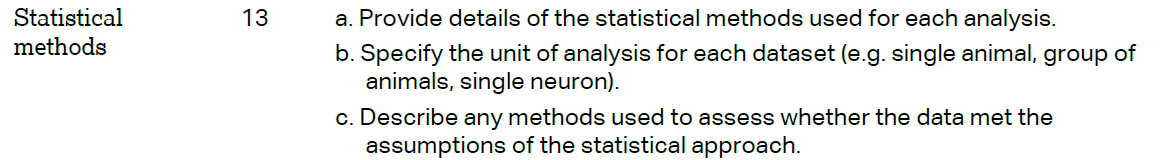 | Section 11 | |
| RESULTS |  | |
| 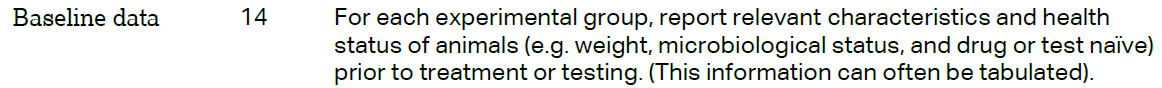 | Figure S2d | |
| 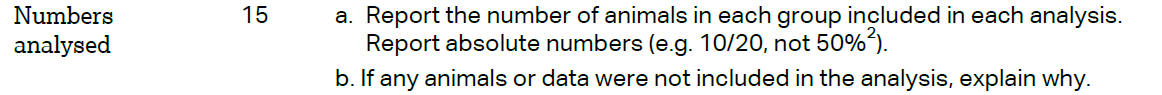 | Paragraph 4 | |
| 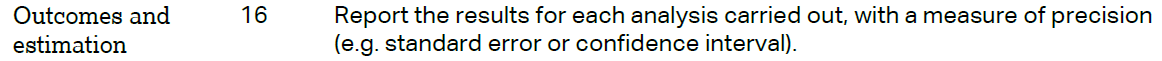 | Figures 4-5 | |
| 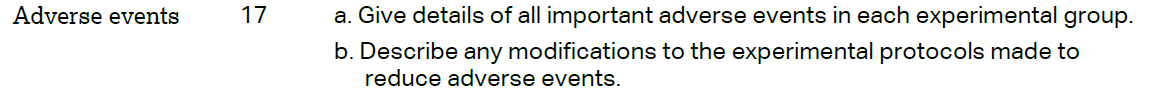 | No adverse events were observed | |
| DISCUSSION |  | |
| 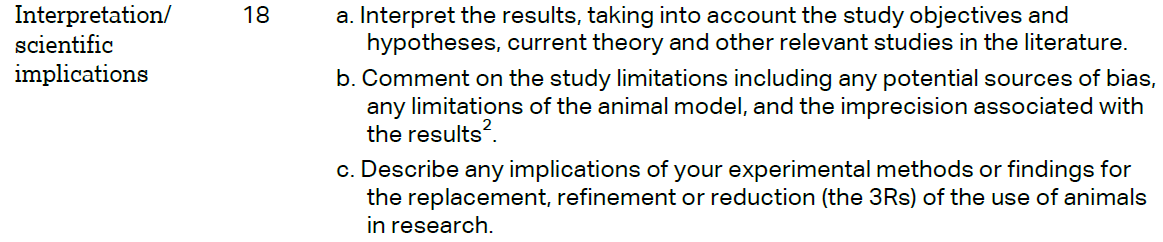 | Discussion  Paragraph 2  Paragraph 1 | |
| 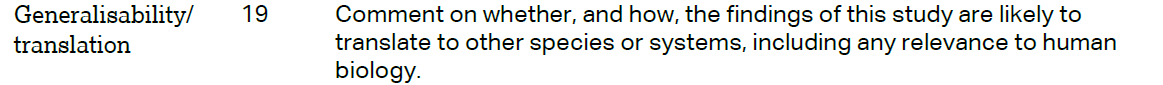 | Paragraph 5 | |
| 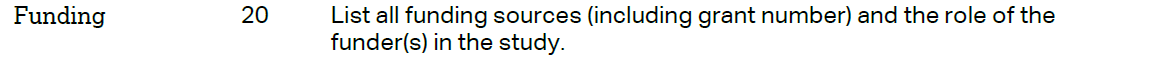 | | NA |


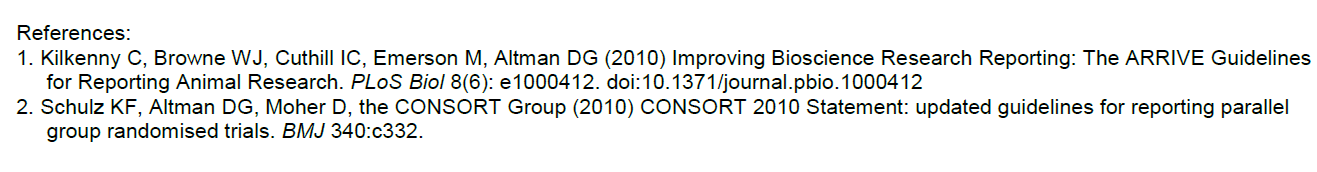

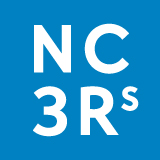

Supplement: S1 ARRIVE Checklist — (DOCX) [file pone.0193289.s001.docx]
