# Supplementary material for: A dystrophic Duchenne mouse model for testing human antisense oligonucleotides
Source: PLoS One. 2018 Feb 21;13(2):e0193289. doi: 10.1371/journal.pone.0193289 (PMC5821388; doi:10.1371/journal.pone.0193289)
Supplement: S1 Table — Primers for generating DNA used for cloning were constructed with an additional restriction side as indicated. a marked primers were used in melting curve analysis. Those primers marked with b were used in multiplex PCR reactions (product size of exon 46, 51 and 52 were respectively 148 bp, 388 bp and 273 bp). The primer set marked by C was developed by Beggs [25] and the d marked set by Chamberlain [24]. (DOCX) [file pone.0193289.s002.docx]

S1 Table: Primers used for PCR reactions.

| **Target** | **Forward primer (5’..3’)** | **Reverse primer (5’..3’)** | **Modification** |
| --- | --- | --- | --- |
| HSV-TK | ccatggccatggggaagcaaaaagcctctcc | ccatggccatggaacacccgtgcgttttattc | NcoI |
| LoxP-blast-LoxP | ctcgagctcgagccctcactaaagggaacaaaag | ctcgagctcgagggccgcttagtttaaactcgag | XhoI |
| intron 51 | gcggccgcgcggccgcatggttttcgatttggccactctat | gcggccgcgcggccgccagggttcttcagcgttgtgtaattc | NotI |
| intron 52 | catatgcatatgtttagcagagcattcctccaccata | catatgcatatgttctggactgggttgaaaaatc | NdeI |
| exon 52^a^ | ggtactccggaatgtctcca | cccagatgacaactaaagaacaaat |  |
| exon 46^b, c^ | gctagaagaacaaaagaatatcttgtc | cttgacttgctcaagcttttcttttag |  |
| exon 51^b, d^ | gaaattggctctttagcttgtgtttc | ggagagtaaagtgattggtggaaaatc |  |
| exon 52^b^ | aaggaatacacaacgctgaaga | cattatttttataaatgtgagggggat |  |
| Intron51-blasticidin | atggtactaaagccacgctaaatttcct | attgtctcatgagcggatacatatttga |  |
| Blasticidin-intron52 | tgtatatcattttactgggggaccttgt | cactaagccctcatttaaaactcctcct |  |
| Exon 47 | TGAAACTGGAGGACCCGTG |  | Human RT-PCR |
| Exon 48 | AAAAGACCTTGGGCAGCTTG |  | Human RT-PCR |
| Exon 49 | aaactgaaatagcagttcaagc |  |  |
| Exon 49 | GATTGAAGTAACAGTTCACGG |  | Murine RT-PCR |
| Exon 55 |  | atcctgtaggacattggcagtt |  |
| Exon 50 | cgtttacttcaagagctgaggg |  |  |
| Exon 50 | GTCTGAGTGGAGGCTGTAAAC |  | Murine RT-PCR |
| Exon 50 | AGGAAGTTAGAAGATCTGAGC |  | Used for sequencing |
| Exon 54 |  | ccaagaggcattgatattctc | Human RT-PCR |
| Exon 54 |  | CGTCTACACTTATCTGCCGTTG | Murine RT-PCR |
| Exon 56 |  | TTGAACAGGGGTGCTTCATC | Murine cDNA |
| Exon 57 |  | TCTGAACTGCTGGAAAGTCG | Human cDNA |
| *Dmd* exon 23 | aaagttctttgaaagagcaa | cagatagttgaagccatttt |  |
| Ex10-16 | ttggaagctcctgaagacaa | gctgtactcttttcaagtttttgga |  |
| Ex11-15 | ggccgggttggtaatattct | ggccagtttttgaagacttgat |  |
| Ex18-26 | tgaatttgcaatctttcggaag | tcttcagcttgtgtcatccat |  |
| Ex19-25 | actgcaagatgccagcagat | ggctgaattgtctgaatatcactg |  |
| Ex26-31 | aagatctatcagagatgcacg | tgtgcaacatcaatctgagac |  |
| Ex27-30 | ctctgcactaggctgaatgg | ctgcgtccaccttgtctgc |  |
| Ex30-36 | agctcaaatgcctcaggaag | tcagcctgaatgatccacttt |  |
| Ex31-35 | ccaatgccatcctggagtt | gggaggtgacagctatccagt |  |
| Ex42-49 | gtccgtgaagaaacgatgatg | cactggctgagtggctgg |  |
| Ex43-48 | cctgtggaaagggtgaagc | gctcttcaaggtcttcaagc |  |
| Ex47-54 | tgaaactggaggacccgtg | ccaagaggcattgatattctc |  |
| Ex48-53 | aaaagaccttgggcagcttg | ctccggttctgaaggtgttc |  |
| Ex57-63 | ccatttggaagccagttctg | tcccagcaagttgtttgagtc |  |
| Ex58-62 | gagtactcttgagactgtacg | actttgtttggcgagatggc |  |
| Ex61-69 | aggaccgagtcaggcagc | ttttatggccttttgcaactc |  |
| Ex62-68 | gccaaacaaagtgccctacta | gttacatttggcctgatgctt |  |
| Ex75 | atgatgcccacctctcc | tgcatcctggcttccaggcg | Biotin-label |
| Ex75 seq | tgctgagctcattgctg |  |  |
| HSK-TK | atgatccagacccacgtcac | ccacacgcgtcaccttaata | Used for sequencing |
| hDMD intron 51 | aacatatttcctgttaaattgttttct | ctaaattgtagcctttctagttaccg | Used for sequencing |
| hDMD intron 52 | catcattcattgctgcaacc | ctaaattgtagcctttctagttaccg | Used for sequencing |
