# Supplementary material for: A dystrophic Duchenne mouse model for testing human antisense oligonucleotides
Source: PLoS One. 2018 Feb 21;13(2):e0193289. doi: 10.1371/journal.pone.0193289 (PMC5821388; doi:10.1371/journal.pone.0193289)
Supplement: S2 Table — (DOCX) [file pone.0193289.s003.docx]

S2 Table: Sequences of TALENs sets and number of chimeric pups that arose from blastocyst injections

| **TALENs set** | **DNA sequence** |
| --- | --- |
| pTAL52E | cgaagtaagttttttaacaagcat |
| pTAL52F | agtgcatgacaagtttcaat |
| pTAL52C_1 | acggatcgaagtaagttttttaacaagcat |
| pTAL52G | gatactaagggatat |
| pTAL52H | ccaaatcctgcattgt |
| pTAL52K | actaagggatatttg |
| pTAL52L | gttccaaatcctgca |

| **ES clone** | **Number of blastocysts** | **Number of pups** | **Ratio m/f** | **Chimera** |
| --- | --- | --- | --- | --- |
| 9B4/1 | 105^a^ | 23 | 11/12 | 4 |
| 10H2/7 | 60 | 7 | 4/3 | 0 |
| 11E7/1 | 65 | 6^b^ | 3/2 | 1 |

*^a^ 3 different injection rounds 20, 45 and 40, ^b^ 1 pup died*
