# Supplementary figures and images for: A dystrophic Duchenne mouse model for testing human antisense oligonucleotides
Source: PLoS One. 2018 Feb 21;13(2):e0193289. doi: 10.1371/journal.pone.0193289 (PMC5821388; doi:10.1371/journal.pone.0193289)

S1 Fig

A

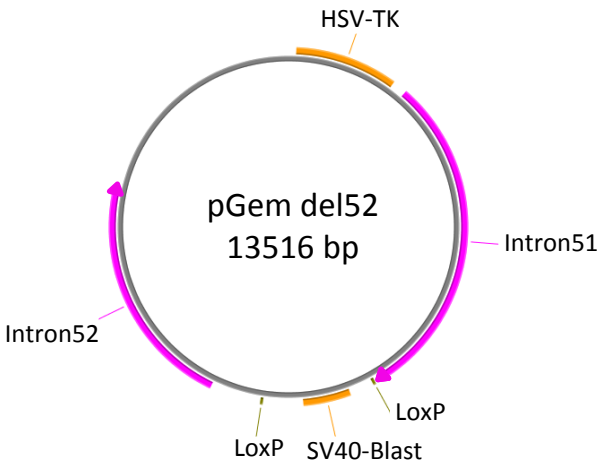

B

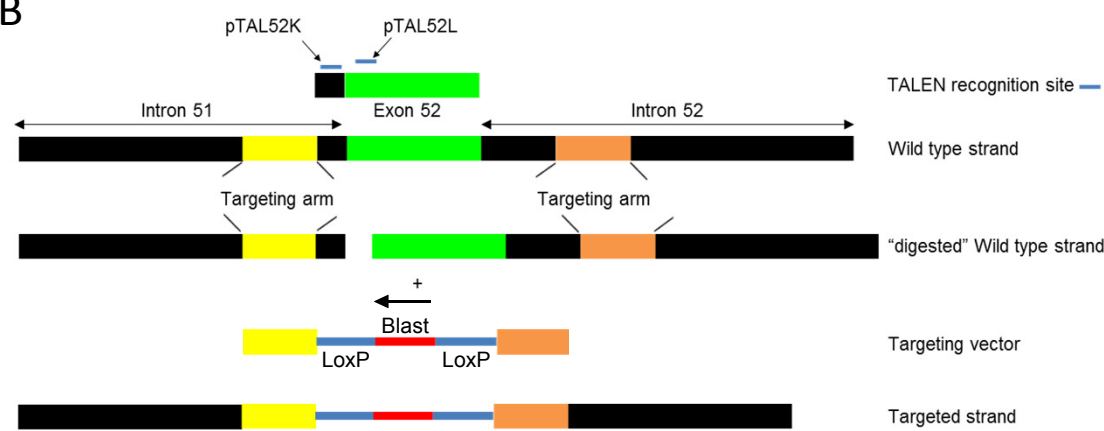

Supplement: S1 Fig — A) Schematic representation of the targeting vector used to delete exon 52 of the hDMD gene. HSV-TK is the negative selection marker, which is placed outside the targeting arm area. I51 and I52 are the targeting arms consisting of autologous hDMD DNA sequence of the intron 51 and 52 obtained by LR-PCR. In between the targeting arms the gene of the positive selection marker blasticidin (blast) is placed, which is flanked by LoxP sites. B) Schematic representation of TALENs facilitated targeting of the hDMD gene. The recognition site of the TALEN set pTal52K/L is at the intron 51 exon 52 boundary and is close to the intron 51 targeting arm. The TALENs induce a double strand break and upon homologous recombination with the targeting vector exon 52 is replaced by the blasticidin gene (NB blasticidin expression is in the opposite direction as dystrophin). (PDF) [file pone.0193289.s004.pdf]

# S2 Fig

A

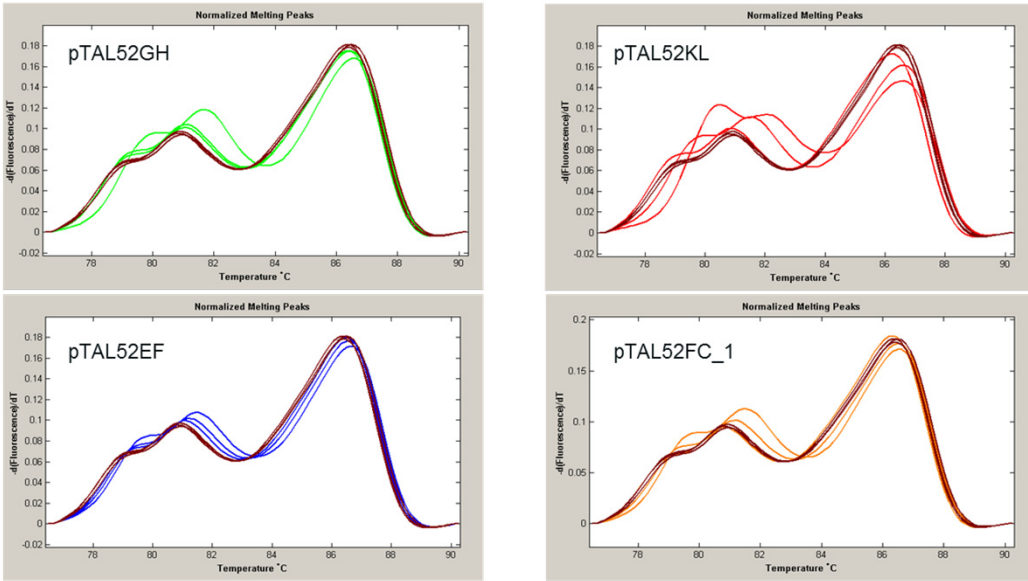

B

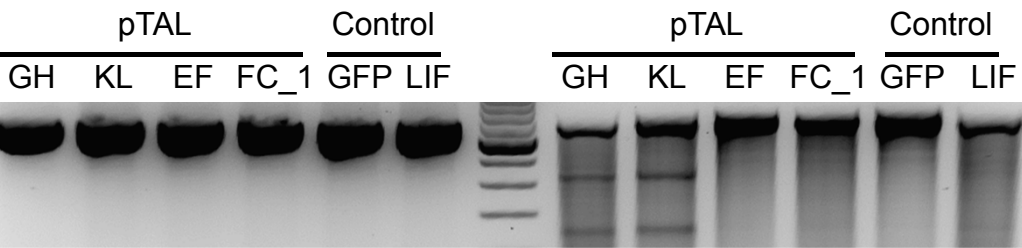

Supplement: S2 Fig — Functionality of the TALENs was determined by analysing DNA of HEK293T cells transfected with the TALENs for TALEN mediated double strand breaks followed by NHEJ. The target area of the TALENs was PCR amplified using melting curve analysis (MCA) A) or B) surveyor assay. The PCR product was analysed for mutations in the sequence, and compared to PCR product utilizing wild type DNA as PCR-template. Data revealed that pTAL-52 GH and KL were most functional with pTAL52KL having the highest functionality (based on MCA). Therefore, the latter set was chosen to facilitate the targeting. (PDF) [file pone.0193289.s005.pdf]

S3 Fig

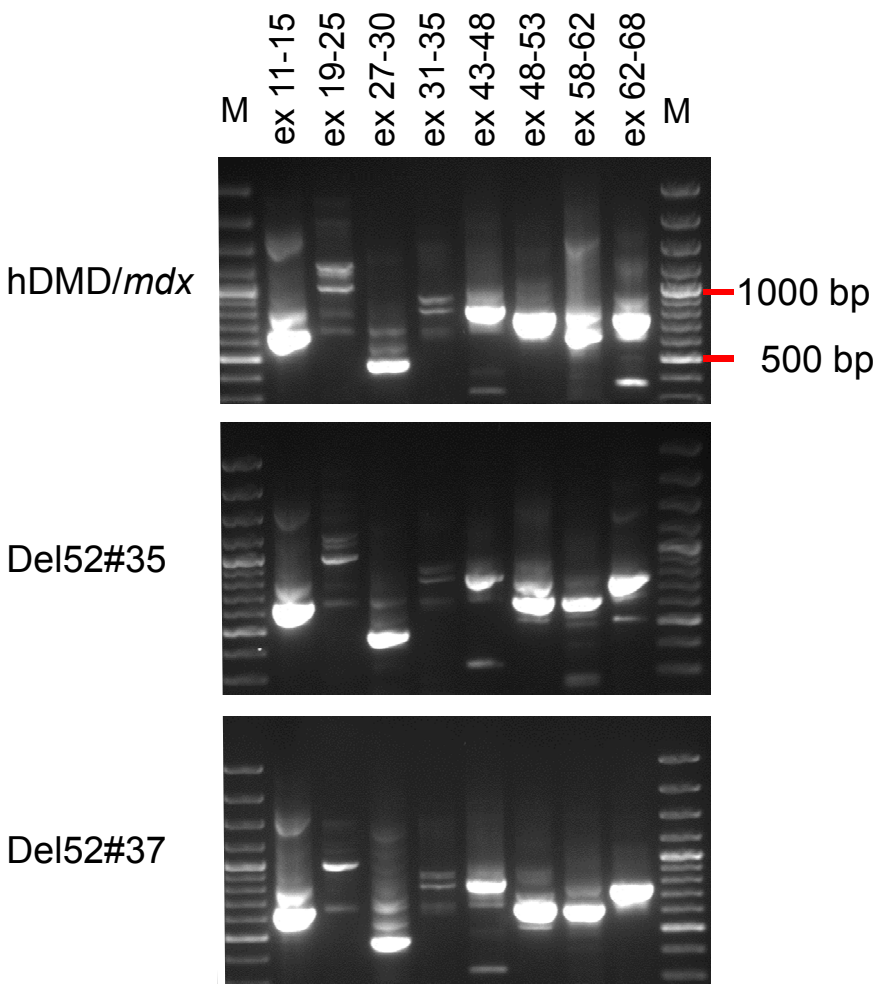

Supplement: S3 Fig — The nested-PCR products have the following sizes: ex 11–15; 633 bp, ex 19–25; 1016 bp, ex 27–30; 463 bp, ex 31–35; 678 bp, ex 43–48; 805 bp, ex 48–53; 735 bp (without exon 52; 617 bp); ex 58–62; 621 bp; ex 62–68; 744 bp. (PDF) [file pone.0193289.s006.pdf]

S4 Fig

A

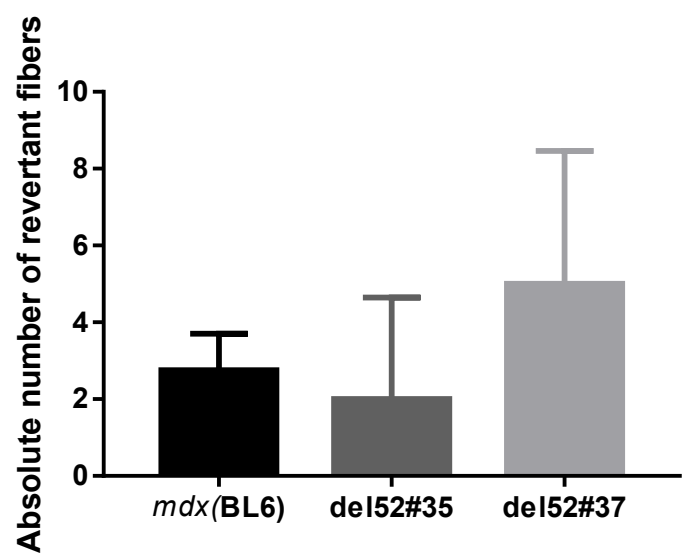

B

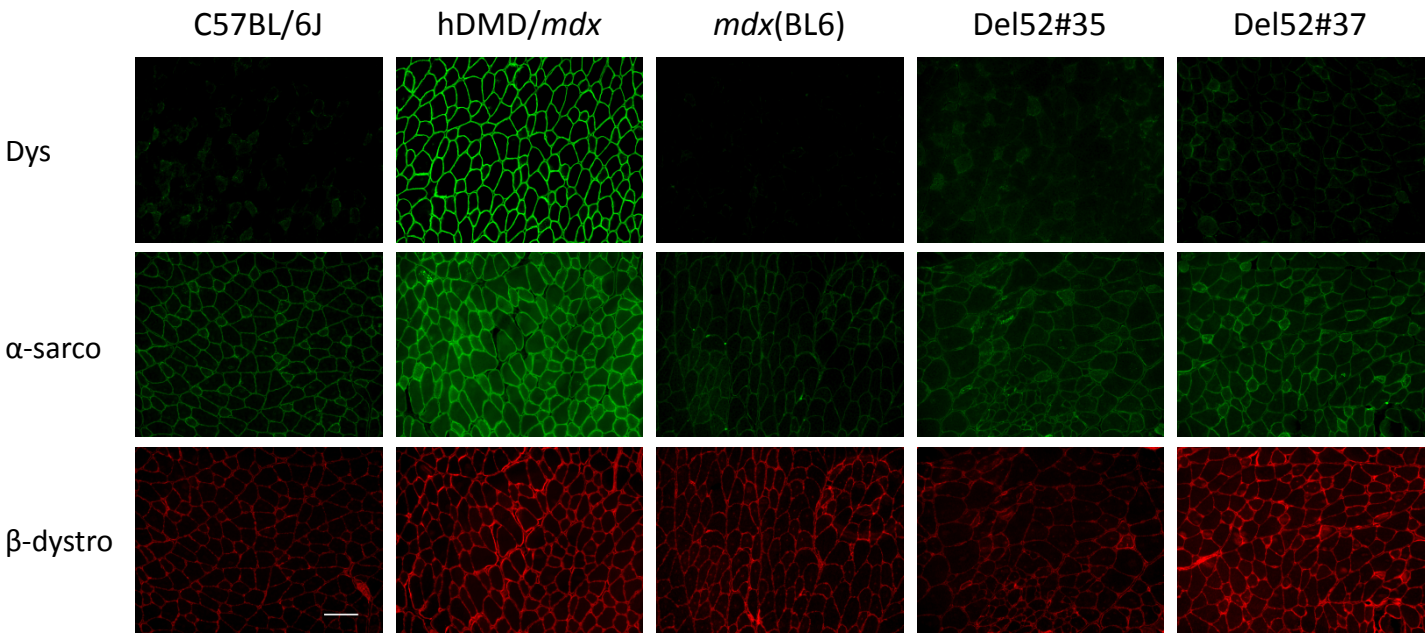

Supplement: S4 Fig — A) The number of revertant fibers, counted on the whole cross-sectional area of the quadriceps muscle, was similar between the three dystrophic mouse strains. B) The quadriceps of all strains stained for dystrophin, α-sarcoglycan and β-dystroglycan. Scale bar is 100 μm. (PDF) [file pone.0193289.s007.pdf]
